# Vmeasur: A software package for experimental and clinical measurement of mesenteric lymphatic contractile function over an extended vessel length

**DOI:** 10.1111/micc.12748

**Published:** 2022-02-10

**Authors:** Peter S. Russell, James J. W. Hucklesby, Jiwon Hong, Enyuan Cao, Natalie L. Trevaskis, Catherine E. Angel, John A. Windsor, Anthony R. J. Phillips

**Affiliations:** ^1^ Applied Surgery and Metabolism Laboratory School of Biological Sciences University of Auckland Auckland New Zealand; ^2^ Department of Surgery Faculty of Medical and Health Sciences Surgical and Translational Research Centre University of Auckland Auckland New Zealand; ^3^ Human Cellular Immunology Group School of Biological Sciences University of Auckland Auckland New Zealand; ^4^ Department of Molecular Medicine and Pathology Faculty of Medical and Health Sciences University of Auckland Auckland New Zealand; ^5^ Drug Delivery, Disposition and Dynamics Monash Institute of Pharmaceutical Sciences Monash University Parkville Vic. Australia

**Keywords:** contractile function, lymphatic, mesentery, microscopy, software

## Abstract

**Objective:**

Conventionally, *in vivo* mesenteric lymphatic contractile function is measured using a high magnification transmission microscope (field of view 0.3–1.5 mm), which precludes visualization of extended lengths of vessels embedded in mesenteric fat. Existing software is not optimized for imaging at a low magnification using a contrast agent. We aimed to develop a simple and clinically transferable method for *in situ* visualization, image analysis, and quantitative assessment of mesenteric lymphatic contractile function over an extended area.

**Methods:**

Subserosal injection of various blue dyes was taken up by mesenteric lymphatics and visualized under a stereomicroscope (25×), allowing for video recording of 1.4 × 1.1 cm of intact mesentery. A new R package (“vmeasur”) that combines multiple high‐performance image analyses into a single workflow was developed. The edges of each vessel were determined by applying an automated threshold to each frame (with real‐time manual verification). The vessel width at every point in each frame was plotted to provide contractile parameters over time and along the lymphatic vessel length.

**Results:**

Contractile parameters and their differences along the length of the vessel were accurately calculated in a rodent model. In a human mesenteric lymphatic, the algorithm was also able to measure changes in diameter over length.

**Conclusion:**

This software offers a low cost, rapid, and accessible method to measure lymphatic contractile function over a wide area, showing differences in contractility along the length of a vessel. Because the presence of mesenteric fat has less of an impact on imaging, due to the use of an exogenous contrast agent, there is potential for clinical application.

**Abbreviations**
AVI: audio video interleave
CSV: comma‐separated values
EDD: end‐diastolic diameter
ESD: end‐systolic diameter
FPS: frames per second
MR: magnetic resonance
NIR: near infrared
OCT: optical coherence tomography
ROI: region of interest

## 1 INTRODUCTION

Lymphatic vessels form a blind‐ended drainage system that recirculates fluid, macromolecules (e.g., proteins, lipids), and immune cells from the interstitial space back into the circulation.^1^ In this way, the lymphatic system has critical roles in supporting body fluid homeostasis and immune regulation.^2^ Mesenteric lymphatics have the additional function of transporting absorbed lipid from the gastrointestinal tract.^3^ Unlike arteries and veins, the mesenteric collecting lymphatics undergo self‐initiated phasic contractions in order to pump lymph fluid toward the central circulation, with backflow prevented by intraluminal bicuspid valves.^4^ These contractions are necessary because lymph frequently drains against a pressure gradient from ^−^4–^−^3 mmHg in the interstitium to ^+^8–^+^12 mmHg in the central veins.^5^, ^6^, ^7^ Phasic contractions of mesenteric lymphatic vessels have been demonstrated in many species (including human, rat, cow, guinea pig), although they are usually weak and infrequently observed in the mouse.^2^, ^8^ Lymphatic contractile dysfunction leads to tissue edema and inflammation,^2^ and has been demonstrated in a wide range of diseases. These include secondary lymphoedema^9^ in humans, and animal models of rheumatoid arthritis,^10^ metabolic syndrome,^11^ obesity^12^ and inflammatory bowel disease,^13^, ^14^ as well as in response to specific inflammatory mediators (e.g., IFNα/IFNβ,^15^ IL‐1α/IL‐1β,^16^ and TNFα^17^) in *ex vivo* isolated lymphatics. Measuring mesenteric lymphatic contractile dysfunction is likely to become increasingly important in both research and clinical settings.

*In vivo* measurement of mesenteric lymphatic contractile function has the advantage over isolated *ex vivo* preparations of retaining the physiological context (i.e., nerves, fat, dendritic cells, and endocrine/paracrine factors).^18^ In general, there are two approaches to *in vivo* imaging: non‐invasive (skin intact) or invasive surgical exposure.^18^, ^19^ Skin intact procedures most commonly involve NIR imaging because of the superior penetrance of this wavelength through tissue. However, spatial resolution rapidly deteriorates beyond 3 mm from the surface,^20^ which means skin intact techniques are not feasible for mesenteric lymphatics, which usually lie deep within the abdominal cavity. It is possible that MR lymphangiography will allow *in vivo*, non‐surgical measurement of mesenteric lymphatic contractions in the future,^21^ but this technology is not yet available for mesenteric lymphatics and would be prohibitively expensive for most laboratories. Therefore, to date, all *in vivo* measurements of mesenteric lymphatic contractility have involved surgical exposure of the mesentery.

The most common technique for imaging mesenteric lymphatics is using an upright transmission microscope to transmit light directly through the mesentery. This process takes advantage of the thin, membrane‐like nature of the mesentery in animal models to directly image short sections of lymphatic vessels without a contrast agent. Transmitted light imaging has a high spatial resolution, leading to accurate and precise measurements of lymphatic contractions, valve movements, and fluid flow (through lymphocyte tracking^22^). This technique has been of immense importance over the last few decades and remains the gold standard for accurate *in vivo* measurement of mesenteric lymphatic contractions. However, because a contrast agent is not used, this approach requires a relatively fat‐free area of mesentery to accurately determine the vessel edge, which usually limits examination to a short section of a single lymphatic vessel (1–2 lymphangions; 1–2 mm).^23^, ^24^ This limited field of view does not allow for measurement of contractile differences along an extended length of lymphatic vessel, making it difficult to obtain a comprehensive overview of mesenteric lymphatic contractile function. While imaging many small regions sequentially can capture some of the variability within the lymphatic tree, this approach is undesirable as it greatly increases the duration of surgery, introducing a time variable to the observations, and it is unable to correlate simultaneous contractions across a section of mesentery. In addition, because of the increased setup time, researchers are often obligated to select vessels undergoing robust spontaneous contractions.^25^ This selectivity, along with the fact that fat‐free mesenteric vessels represent the minority of vessels, can create a selection bias in sampling when comparing groups. The clinical transferability is also limited, as human mesentery is too thick to allow for imaging using transmitted light, and even if this could be overcome, positioning human mesentery on a microscope stage is logistically challenging. A novel technique using Doppler OCT has been developed,^26^ which has the major advantage of not requiring a contrast agent, but similarly, the optical setup is complex and its performance over wide fields of view for vessels embedded in mesenteric fat remains unclear.

Stereomicroscopy involves illuminating the sample from above and visualizing the light reflected off the sample, which drastically increases the area that can be imaged. Transmitted light is not required, so the sample does not need to be positioned on a microscope stage, solving many logistical problems. As stereomicroscopes allow for 3D visualization, and for the user to work under standard lighting conditions, they are already ubiquitous in surgical environments. Introducing a tracer dye to the vessel provides a high level of contrast, even in human vessels covered by moderate quantities of mesenteric fat. However, to the best of our knowledge, a software platform for measuring extended lengths of mesenteric lymphatic vessel using a contrast agent has not been developed because of the reliance on non‐contrast transmitted light microscopy. Currently available software for tracer dye‐contrasted vessels has been optimized for other tissue beds, such as mice popliteal lymphatics, and does not adequately determine differences in contractility over the length of a vessel.^19^, ^27^, ^28^, ^29^ Existing software is also not optimized for the challenge of vessels embedded in mesenteric fat, which limits the potential for measuring human mesenteric lymphatic contractility.

Thus, there remains a need for a readily accessible software platform to measure mesenteric lymphatic contractile function over extended lengths of vessels using simple microscopy techniques. In this study, we aimed to develop a simple and clinically transferable method for analyzing stereomicroscopic data, allowing straightforward quantification of the lymphatic vasculature over a wide field of view.

## 2 METHODS

### 2.1 Animals

Male Sprague‐Dawley rats (350–500 g), bred by the University of Auckland animal facility, were fed a standard rodent chow diet *ad libitum* and housed in a humidified room with a 12:12 h light:dark cycle. All rodent experiments were approved by the University of Auckland Animal Ethics Committee.

### 2.2 Rodent surgical preparation

General anesthesia was induced in an induction chamber with isoflurane (MedSource, New Zealand) (4% in 3 L/min O_2_). The rat was then transferred to a nose cone under isoflurane (4% in 40% O_2_/air mix). It was placed on a RightTemp^®^ heating pad with rectal temperature probe (Kent Scientific Corporation, Torrington, CT, USA) to maintain body temperature at 37°C. Subcutaneous Temgesic^®^ (0.1 ml/100 g BW; 300 µg/ml buprenorphine, Reckitt Benckiser, Berkshire, England) was injected for analgesia. Betadine^®^ (7.5% povidine‐iodine, Sanofi‐Aventis, Queensland, Australia) was used prior to skin incisions. A tracheostomy was performed (using a modified 14G angiocath, BD, Utah, USA) and connected to a Rovent^®^ Small Animal Ventilator (Kent Scientific Corporation, Torrington, CT, USA). The animal was ventilated on volume priority with 1.5%–2% isoflurane (40% O_2_/air mix). Expired CO_2_ was maintained at 35–45 mmHg and measured by an EMCO Capnograph 3400 (EMCO Meditek Pvt. Ltd., Mumbai, India). Maintenance fluid (0.9% saline) was infused into the femoral vein at 6 ml/kg/h.

**FIGURE 1:**
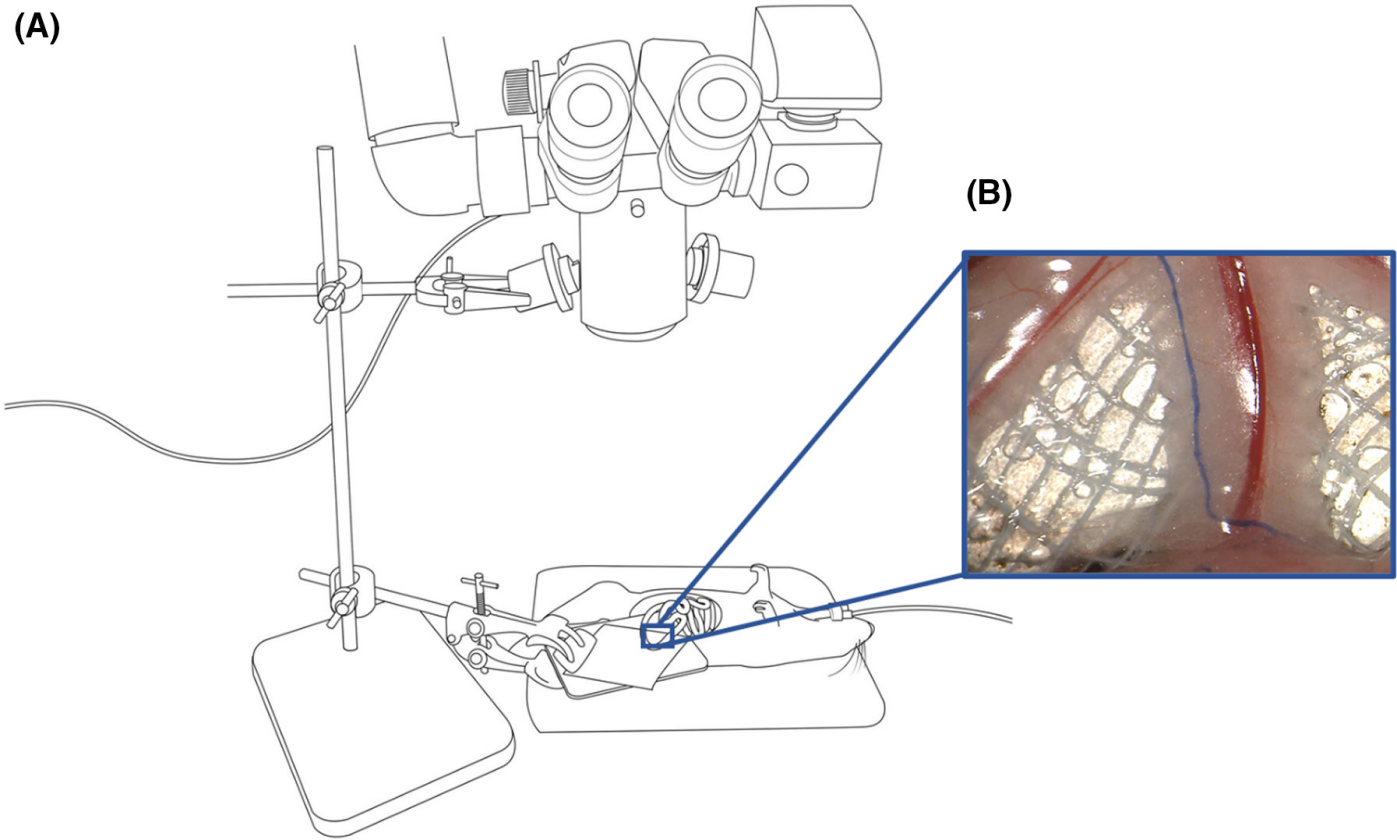
Surgical setup for mesenteric imaging. (A) The rat was anesthetized and positioned under a dissecting microscope. A loop of jejunum with attached mesentery was exposed on a piece of moist gauze supported on a platform for imaging. (B) After subserosal injection of blue dye, the mesenteric lymphatic vessel is immediately visible. Dimensions of the blue rectangle are 1.4 × 1.1 cm; the bowel wall is at the lower border of the image, and the root of the mesentery is just out of view at the top of the image

A longitudinal or transverse laparotomy was performed. A loop of jejunum 5 cm from the duodenojejunal flexure (although any section of jejunum or ileum can be used) was exteriorized and laid atop a piece of white moistened gauze on a horizontal platform attached to a retort stand, taking care to ensure the loop was handled gently and maintained, without tension, at the same height as the root of the mesentery (Figure 1A). The bowel loop and mesentery were moistened with drops of warmed 0.9% saline immediately prior to dye injection. The bowel wall was visualized with a Leica M220 F12 Surgical microscope (Leica Microsystems, Wetzlar, Germany) at 25× magnification with in‐built white LED illumination. The microscope image was captured at a right angle to the plane of the mesentery.

A blue dye (either 1% w/v Evans Blue (Aldrich Chem. Co., Milwaukee, Wisconsin, USA), 0.4% w/v Hyclone Trypan Blue (Gibco, Thermo Fisher Scientific, Waltham, Massachusetts, USA), or 2.5% w/v Patent Blue VF (Acros Organics, New Jersey, USA) in 0.9% saline) was carefully injected sub‐serosally into the antimesenteric border of the bowel wall using a 30G needle (BD, Utah, USA) and 1 ml syringe (Terumo, Leuven, Belgium). The dye was injected into a Peyer's patch, if one was available, as this was technically easier and caused the dye to stay in the lymphatics longer. The needle was advanced into the bowel wall at an acute angle to the surface so that it was visible just under the serosal surface. Serosal lymphatics were seen to fill during the injection if the needle tip was in the right plane. Enough dye was injected to completely fill the draining mesenteric lymphatic(s), which usually required approximately 10 µl and never exceeded 30 µl. Once the mesenteric lymphatics were filled with blue dye (Figure 1B), the microscope was centered over the mesentery, giving a viewing area of 1.4 × 1.1 cm. The microscope was stabilized with a clamp attached to the retort stand, and a video (24 bits per pixel color, 1024 (W) × 768 (H) pixels, 22 FPS) was recorded using a Leica MC190 HD microscope camera (Leica Microsystems, Wetzlar, Germany) and the accompanying LAS EZ software (v3.4.0, Leica Microsystems, Switzerland) and stored as an AVI file (Video S1). This process was repeated in different sections of the bowel and mesentery. After injection, the lymphatics typically remained blue for 10–20 min but longer recordings were possible with re‐injection, continuous subserosal infusion, or by injection into Peyer's patches. The mesenteric lymphatic imaging technique was progressively developed over a series of five rats, then applied and further refined in various other studies in our laboratory that have used the technique to generate their key lymphatic function output results. All rats were euthanized under anesthesia at the completion of the surgery.

### 2.3 Data analysis

All computational analysis was conducted using a custom R package vmeasur (vmeasur, V0.1.3) (available on the Comprehensive R Archive Network (CRAN) at www.cran.r‐project.org/package=vmeasur). vmeasur was run using R (Version 4.1.0)^30^ in conjunction with RStudio (Boston, MA, USA, Version 1.4.1106). It uses functions derived from a variety of specialist pre‐existing R packages (discussed below) to provide a single easy‐to‐follow workflow; however, the user does not need to interact with any of these packages directly. Functions from the imager package were used to manipulate images and provide interactive graphical user interface elements. Where the processing of video files was required, the av package was used. Parallel processing was achieved with components from the futures package, and progress bars were generated with progressr. The packages tidyr, dplyr, and stringr were used to handle the data produced, while contraction minima were detected with pracma. All graphs were generated with ggplot2 and ggpubr. Pixel size was calibrated using an image of a ruler graded in mm and the calibrate_image function (Figure S1). In our setup, each pixel was 13.7 µM in size.

### 2.4 Specifying regions of interest to isolate individual lymphatic segments

**FIGURE 2:**
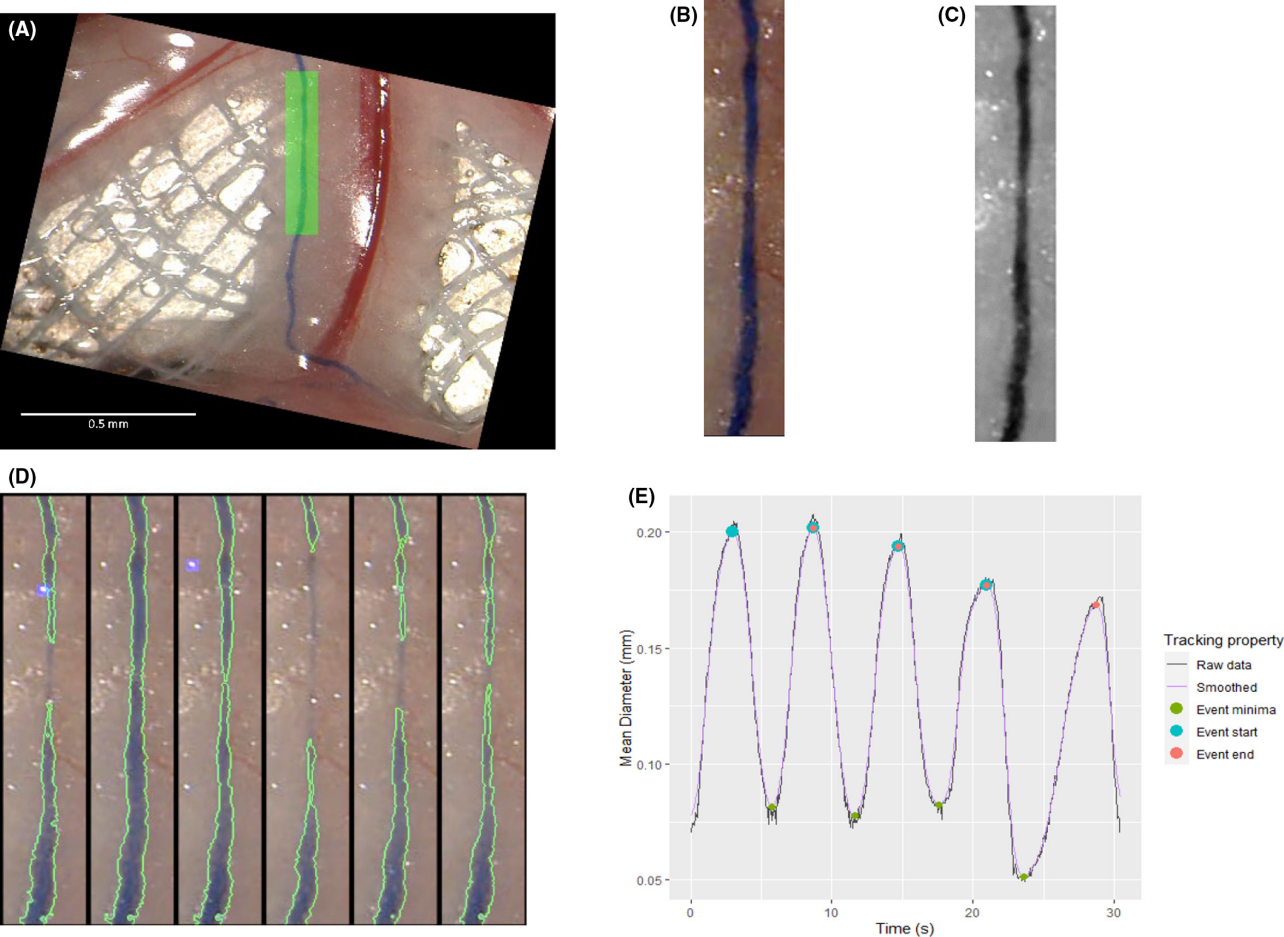
vmeasur isolates regions of interest (ROI) and segments lymphatic vessels. (A) The *select_roi* function in vmeasur was used to graphically select a section of lymphatic vessel, shown highlighted in green. The bowel is at the lower border of the image. (B) The region of interest was isolated and rotated so that the vessel is positioned vertically. (C) Red channel of the rotated image, clearly showing the lymphatic vessel. (D) vmeasur segmentation of six equally spaced frames from the video with the edges of the lymphatic vessel superimposed in green, and overexposed pixels highlighted in purple. (E) Mean diameter of the region of interest in each frame, with the calculated tracking properties superimposed

In order to quantify lymphatic contractility, a ROI encompassing one section of vessel was specified. The width, length, and rotation of the ROI varied as the area selected had to isolate a single lymphatic section, avoiding branch points, blood vessels, and areas containing imaging artifacts (green section, Figure 2A). While not shown here, ROI selection can be repeated within any one video to simultaneously measure multiple sections of the vascular tree.

The ROI was defined using the select_roi function, which prompted the user to select the video file for analysis. The first frame from the video selected was then displayed, and the user was prompted to define the orientation and extent of the ROI by dragging over the image. The ROI was then overlaid in green and displayed to the user for confirmation (Figure 2A). The ROI parameters were then applied to every frame to produce a video containing only the area encompassed by the ROI, with the lymphatic vessel vertically oriented within the frame (Video S2). While this isolation and rotation process is computationally intensive, vmeasur was able to accomplish this task in several seconds on a moderately high‐end CPU (such as the Intel Xenon Gold 6254).

### 2.5 Vessel segmentation

The edge of the vessel was determined by applying a threshold value to the red channel of the video (as the wavelengths detected as red by a standard camera align with those absorbed by the blue dye [Figure 2C; Figure S2]). As the ROI varied substantially in contrast within and across animals due to anatomical variation, an absolute threshold was unable to detect vessels reliably. Therefore, the threshold function from the imager package was applied to six frames from each ROI, and the mean of the six thresholds calculated was used as the default region for all frames of the video. Any pixel below this threshold was determined to be lymphatic, while any above was not. Following thresholding, vmeasur excluded any areas determined to be lymphatic that were less than 10 px in area (137 µM^2^); these regions were the result of imaging artifacts and too small to be viable lymphatic vessels.

Light reflection inevitably occurred, making it difficult to see the underlying lymphatics in some areas. These reflections appeared as bright white spots that saturated all color channels of the image (evident in Figure 2B,C). Due to their high intensity, these spots were not detected as lymphatic. Hence if a spot overlaid a lymphatic vessel, the affected section would appear narrower than it was. Therefore, we excluded these sections of vessel from later analysis. To ensure the entire affected area was removed, any sections of the vessel closer than 5 pixels from a saturated pixel (determined to have a value >99% of the camera's range) were automatically excluded from subsequent measurements. The entire excluded area was displayed to the user as a purple highlight (Figure 2D). To ensure that the threshold was accurate across the duration of each video, six equally spaced frames across the length of the video with the calculated vessel boundaries and excluded areas superimposed were automatically displayed (Figure 2D). The user was then prompted to either confirm this threshold or manually adjust the value as required. Manual adjustment of the threshold was usually required in the presence of dye leak in diseased animals or for poorly visible lymphatics in areas high in mesenteric fat.

After accepting the threshold, vmeasur applied the segmentation algorithm to all frames of the video, and then saved a CSV file containing the width of the vessel in each row of pixels in each frame and an AVI video showing the original images overlaid with the calculated vessel boundary and any saturated areas excluded (Video S2). The latter file allowed the user to manually inspect the calculated vessel boundaries and ensure they aligned with the original images. Finally, a line of code containing all the parameters selected was printed to the console, allowing the analysis to be reproduced precisely. Furthermore, by modifying and re‐running this code, the user could manually fine‐tune the tracking parameters or apply the same settings to multiple videos.

### 2.6 Contraction event detection and quantitative measurement

The quantify_mean_width function in vmeasur was used to plot the mean width of the vessel within the ROI over time (Figure 2E, gray line). To improve image noise, vmeasur applied the kmeans algorithm to the series of widths to smooth out any small aberrations (Figure 2E, purple line). The window used for smoothing was 20 frames (0.88 s).

**FIGURE 3:**
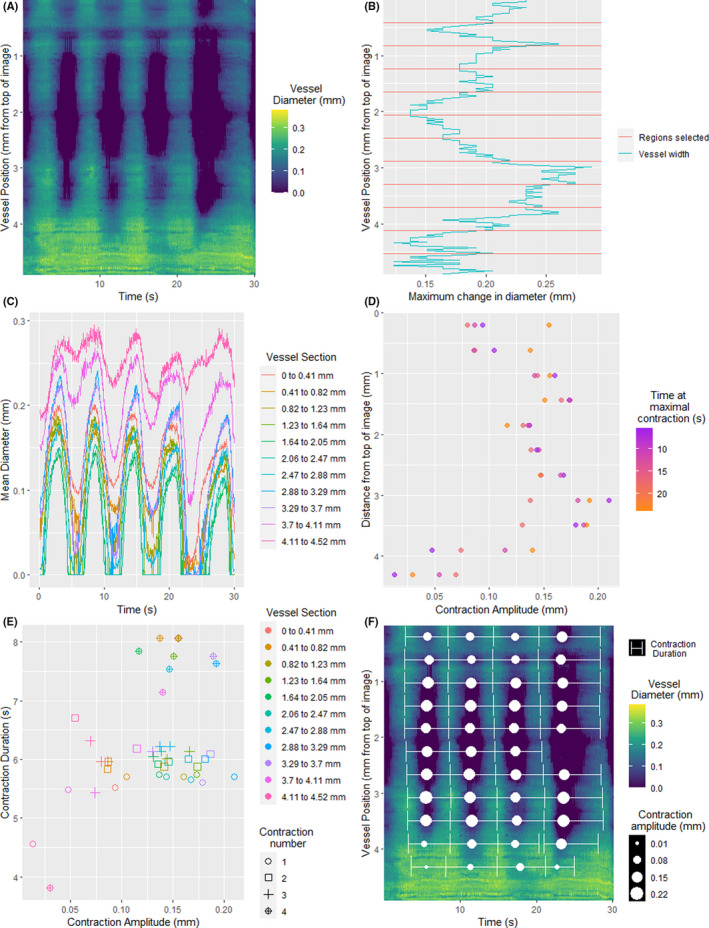
Computational analysis for quantification of lymphatic function along the vessel length. (A) The width of the vessel was calculated at every horizontal line of pixels in each frame and plotted as a heatmap. (B) The maximum change in diameter (peak – nadir) throughout the course of the video varied substantially along the vessel length. Horizontal red lines demarcate the 30‐pixel (0.41 mm long) sections. (C) When divided into 30‐pixel sections, substantial differences in contraction amplitude, but not contraction frequency, are seen across the vessel. (D) Contraction detection was performed on each section of vessel, showing that the amplitude of each contraction varied over time and along the length of the vessel. (E) Calculated contraction amplitudes for each contraction in each section of vessel were plotted against the total duration of the respective contraction. (F) The calculated duration of each contraction and its amplitude are presented overlaid on the original heat map for manual review. Note that a contraction was not detected during the last part of the video for the section approximately 2.5 mm from the top of the image because the diameter movement was less than 1 pixel (i.e., the vessel was already nearly fully contracted)

To investigate the differences in contractility along the vessel length, a variety of vmeasur functions were used to analyze smaller sections individually. Firstly, the quantify_width_position function was used to generate an overall heat map of the vessel's diameter over time and a plot showing the maximum change in vessel width at each position (Figure 3A,B). Based on this plot, the vessel was divided into sections for further analysis. The sections were selected so that they were large enough to average out random brightness fluctuations affecting single pixels, while remaining small enough to capture the variation over the entire vessel length. For our study, these segments were 30 pixels long (0.41 mm).

Both the vessels overall, and each section individually, were analyzed using the quantify_mean_width function to detect contraction events. A contraction event encapsulated one cycle of contraction and re‐filling. The first step of this process involved identifying the minima (troughs). By default, a minimum was detected if the proceeding 10 frames were all decreasing, and the following 10 frames were all increasing. Once detected, each minimum was analyzed to determine the full extent of the contraction event. Algorithmically, this was achieved by continuing to look forwards through the diameters measured in each frame until the curve flattened or began to decrease, and this point was determined to be the end of the contraction event. By repeating this process looking backward through measured vessel diameters, the start of the contraction event was detected. Events where the average diameter decreased by less than one pixel, and those that lasted more than 110 frames (5 s) were excluded from further analysis, as these were too small or long lasting to be a viable lymphatic contraction.^4^ While these parameters were optimal for our datasets, vmeasur allows for customization of the number of frames used in each step. Once all the contraction events were detected, the quantify_mean_width function automatically derived several lymphatic contractile parameters (Table 1, Figure 3).

**TABLE 1:** Contractile parameters derived from Video S1

Aspect of Wave From	Description or Formula	Units	Mean ± SD^a^
No. of contractions	No. of contractions in the video	absolute	4
Contraction frequency (CF)	1/(average length between contractions)	min^−1^	9.43
End‐diastolic diameter (EDD)	Diameter immediately before contraction	mm	0.20 ± 0.01
End‐systolic diameter (ESD)	Diameter immediately after contraction	mm	0.08 ± 0.02
Contraction amplitude (CA)	EDD −ESD	mm	0.12 ± 0.01
Ejection fraction (EF)	(EDD^2^ − ESD^2^)/EDD^2^ × 100	%	84.68 ± 4.45
Fractional pump flow (FPF)	EF × CF	% min^−1^	798.93 ± 41.99
Peak	Widest diameter, whether associated with contraction or not	mm	0.20 ± 0.01
Nadir	Smallest diameter, whether associated with contraction or not	mm	0.08 ± 0.02
Contraction duration	Time from EDD to ESD	s	2.75 ± 0.13
Muscle shortening speed	CA/contraction duration	mm s^−1^	0.04 ± 0.00
Relaxation duration	Time from ESD to next EDD	sec	3.61 ± 1.00
Filling speed	(EDD – previous ESD)/relaxation duration	mm s^−1^	0.03 ± 0.01
Percent re‐filling	(EDD – previous ESD)/previous CA ×100	%	93.44 ± 6.92

Note

Values were taken from the entire ROI (Video S2) and averaged (±SD) across the four separate contraction events in the video.

^a^ Calculated across the four consecutive contractions observed in the ROI shown in Figure 2E.

### 2.7 Human study

An image of a dye‐infused human mesenteric lymphatic was collected during a separate study approved by the University of Auckland Health and Disability Research Committee (Protocol number 17/NTA/249). The patient provided written consent and confirmed approval for use of the image in this paper. The intra‐operative image was taken on a DSC‐RX100 Digital Compact Camera (Sony, New Zealand) as part of an ongoing mesenteric lymphatic architecture study. This image came from a separate clinical anatomical study that did not include videos of contractions but did supply a still image of the dye path that we could use for the software analysis. The maximal vessel size was estimated from the clinical observation to be approximately 1 mm. The image was taken approximately 30 min after subserosal injection of 1–2 ml of 2.5% w/v Patent Blue V (Guerbert, Paris, France) in 0.9% saline. The dye was administered via a 25G needle into a normal piece of the jejunum (halfway between the mesenteric and antimesenteric border) during a standard open abdominal procedure. Image processing was identical to that used in the rodent study; however, as only a single image required analysis, the threshold_vessel function in vmeasur was used in place of the select_roi function.

## 3 RESULTS

Analysis of an example video (Video S1) is shown in Figures 2 and 3, Video S2, and Table 1. The vessel shown is highly contractile, changing between an average of 82 and 205 µm in mean diameter over a 30 s period (Figure 2E). The different contractile parameters measured from this vessel are listed and defined in Table 1.

### 3.1 Lymphatic contractions varied along the vessel length

The parameters detailed in Table 1 were calculated from the average vessel diameter from the entire ROI. While this provides quantitative data of the overall vessel contractions, it is unable to give an appreciation of contraction variability along the length of the vessel. Therefore, we carried out further analytics to investigate the level of consistency in contractions along the vessel length.

We found that the lymphatic contractions were not consistent along the length of the vessel, which could be visualized using a heatmap (Figure 3A). The diameter of each location over time can be visualized by looking left to right across the heatmap, while the diameter at various locations can be examined by looking up and down. This heatmap demonstrates that there was marked heterogeneity in diameter change along the length of this vessel. To visualize the extent of heterogeneity, the maximum difference in diameter (peak – nadir) is presented in Figure 3B. While some sections underwent maximum diameter changes of up to 0.28 mm, others remained almost static. Figure 3B shows that the section between 3 and 3.25 mm from the top of the image underwent the largest overall change in diameter, and the section between 4.25 and 4.75 mm underwent the smallest change.

To further quantify this heterogeneity, each 30‐pixel section was analyzed individually. Figure 3C shows the average diameter of each 30‐pixel section over time. All sections appeared to contract at a similar time, giving the same contraction frequency, but the contraction amplitude differed markedly along the vessel.

Contraction data could also be obtained on a per‐contraction basis. Figure 3D presents the amplitude of each contraction in each section of vessel. The central section of the vessel showed large contraction amplitude with little variability between contractions, whereas the sections of the vessel that had small contraction amplitudes showed more variability. Figure 3E shows the relationship between contraction amplitude and contraction duration for each individual contraction. Here, we see that the last contraction in the video had a longer duration for a similar amplitude across the vessel length (likely representing a slower muscle shortening speed) when compared to the other contractions. Overall, these graphs highlight the wealth of contractile data that can be gathered by simultaneously monitoring a substantial length of lymphatic vessel.

### 3.2 Validation of contraction event detection shows that vmeasur can accurately detect contractions

The accuracy of the contractile parameters is highly reliant on correct contraction detection. To validate this, the tracked parameters were superimposed on the heatmap (Figure 3F). In this figure, the timepoint at which the ESD is reached is presented as a dot, the size of which is determined by the contraction amplitude. Horizontal bars are used to show the time from one EDD to the next. This overlaid data closely correlates with the heatmap, and when combined with visual inspection of Video S2, this confirms that vmeasur correctly detected the contractions.

### 3.3 Applying vmeasur analysis to clinical applications

**FIGURE 4:**
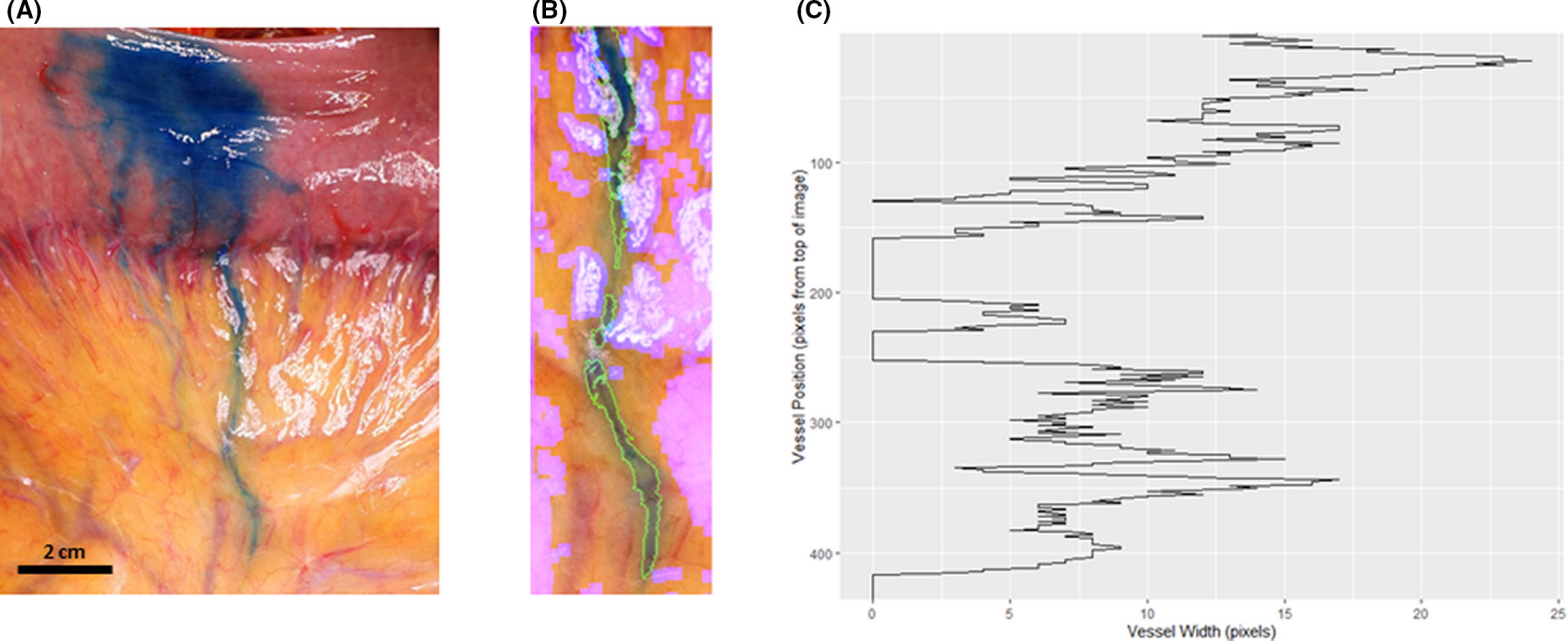
vmeasur can quantify lymphatic vessel diameter in human mesentery. Human jejunum was sub‐serosally injected with patent blue. (A) An image of the intestine and mesentery showing dye uptake by a mesenteric lymphatic. Image taken 30 min following dye injection. (B) The main lymphatic vessel was segmented with vmeasur. White and pink areas represent areas detected as light glare. (C) The calculated diameter along the length of the lymphatic vessel (given in pixels as no calibration was available)

In a separate human study, patent blue dye was sub‐serosally injected into the jejunum during open abdominal surgery, and taken up by the mesenteric lymphatics (Figure 4A). Segmentation of a single representative image using vmeasur successfully detected the lymphatic vessel (Figure 4B). Subsequent image analysis was used to calculate the vessel diameter along its length (Figure 4C). In this pilot study, no video recording was available and so change in diameter over time could not be calculated. However, based on the successful vessel detection in this example and the rodent model presented above, vmeasur analysis of human video data would also be feasible in the clinical setting.

## 4 DISCUSSION

In this paper, we have described a simple method for quantifying rodent mesenteric lymphatic contractility over a wide area of mesentery using a new R package. We have also used the method to analyze a dye‐filled mesenteric lymphatic from an intra‐operative human image. The method is compatible with a range of commercially available blue dyes (Figure S2) and could be readily adapted for fluorescent imaging if desired. It makes use of a commonly used surgical stereomicroscope. Nothing precludes the use of this software package in other species, or where the vessel is obscured by moderate quantities of mesenteric fat. Due to the wide field of view, the relatively poor visibility that can result from overlying mesenteric fat is less important, as the average diameter over a long vessel will approach the true diameter.

**FIGURE 5:**
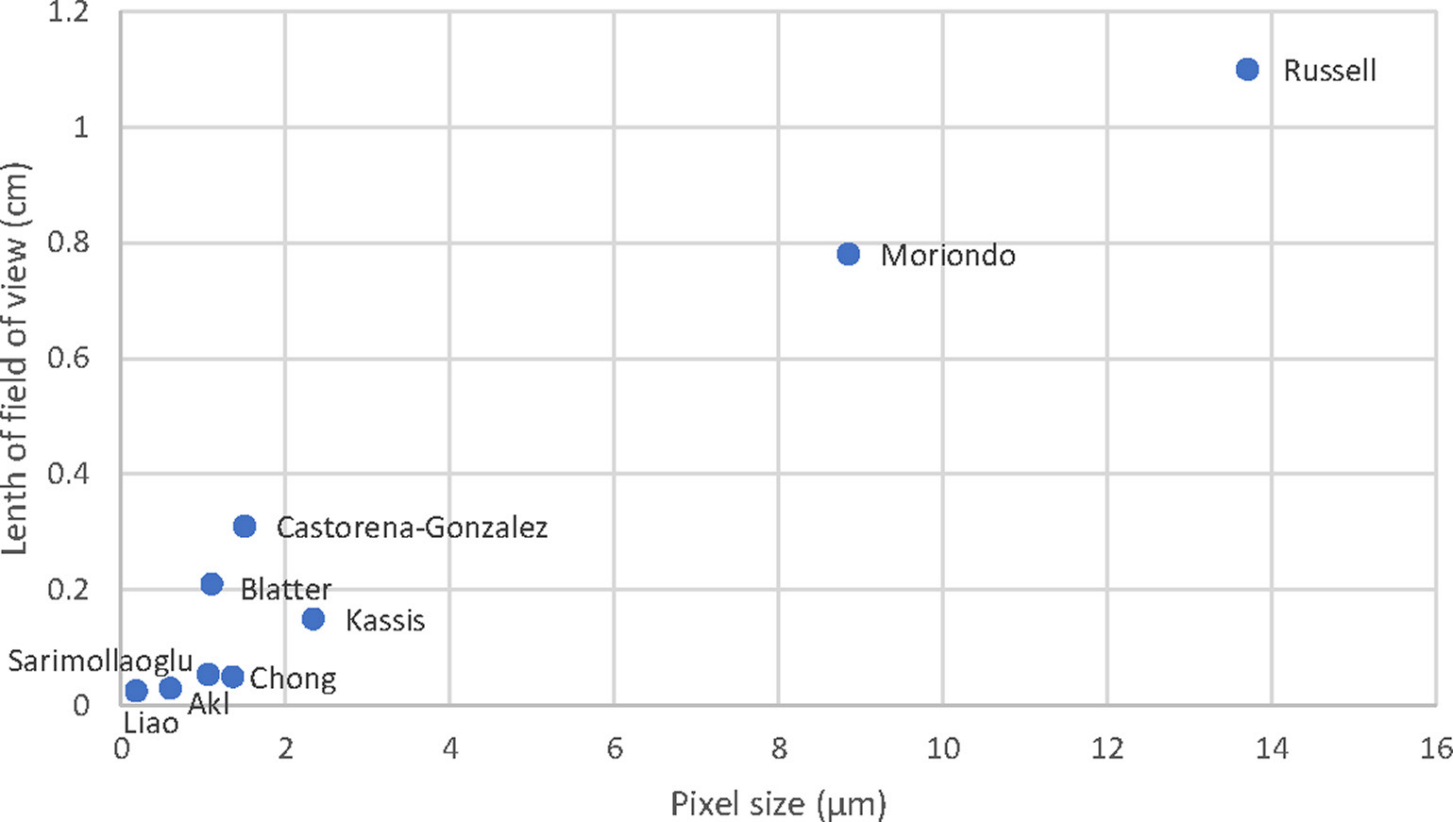
Comparison of the previous studies summarized in Table 2 to demonstrate the trade‐off between field of view and pixel size (to give an estimation of spatial resolution). The studies in the figure are labeled by the publication's first author's name. For Blatter et al.,^26^ the data point represents the resolution of the optical coherence tomography setup presented. Please note that our pixel size of 13.7 µm can be improved with a higher resolution camera or by using a higher magnification optical components with reduced area; hence, this datapoint is not an inherent feature of the software package vmeasur

We compared the key features of this method to other recent *in vivo* lymphatic image analysis software platforms in Table 2 (methodologies included for completeness). Figure 5 gives a graphical comparison of the imaging specifications (field of view and resolution [pixel size]) of the studies from Table 2. The primary advantage of this technique is the wide field of view (1.1 cm, compared to 0.3–1.3 mm for standard high magnification methods [Table 2]), which is the result of using low power magnification with a contrast agent that better delineates vessels that are embedded in mesenteric fat. While the use of a contrast agent for visualizing mesenteric lymphatics is far from new, we have been unable to find an existing software package that is easy to use and can adequately quantify these data. Software for measuring lymphatic contractility under both transmitted light and fluorescence microscopy of the lymphatics has been described.^28^, ^29^, ^31^ This existing software uses automated edge detection at manually selected single points and thereafter measures diameter over time at that single point. This technique is inadequate for vessels embedded in mesenteric fat where the visibility at a single point may fluctuate over time, or where studies of extended lengths of the vasculature are required. Instead, continuous diameter measurements along the vessel are required so that deterioration in visibility can be compensated for by measuring over a much longer length. By recording continuous diameter measurements and dividing the vessel into multiple sections, any variations in mesenteric fat or noise in the signal within each section are averaged out, while heterogeneity in the contraction throughout the vessel can still be measured. Castorena‐Gonzalez et al.^27^ described a technique that measures continuous fluorescence intensity along the length of a mouse popliteal lymphatic, but it is unknown if fluorescence intensity closely correlates with change in diameter in the presence of mesenteric fat.

**TABLE 2:** Comparison of our method to other recent *in vivo* surgical methods for evaluation of lymphatic contractile parameters

Authors	Model	Contrast	Software Platform	Segmentation/Edge detection	Requires minimal/no mesenteric fat	Image processing outputs	Quantification outputs	Field of View	Spatial resolution
*Stereomicroscopy with blue tracer dye*									
Russell et al. (2021)	Rat mesenteric lymphatics	Injection of blue dye into bowel wall	R, open source, deposited in a public R repository	Automated blue channel thresholding across the whole vessel with manual validation available	No	‐ Diameter over time ‐ Contraction frequency	Continuous diameter along the length, but separable into sections	1.4 × 1.1 cm	Pixel size 13.7 µm; 1024 × 768 pixels
*Stereomicroscopy with fluorescent tracer*									
Castorena‐Gonzalez et al. (2018)^27^	Mouse popliteal lymphatic	Injection of 2% FITC‐dextran into footpad	Custom Python‐based programs	Fluorescence intensity at each point along vessel	N/A	‐ Fluorescence intensity over time	Continuous fluorescence intensity along length	~3.1 mm visualized at one time	2048 × 2048 pixels
Chong et al. (2016)^29^	Mouse flank lymphatic	Infusion of P20D680 dye into inguinal LN	Axiovision/Excel/Custom MATLAB scripts (Scholkmann et al. 2012)	Single point manually drawn ROIs; mean signal intensity of vessel ROI divided by mean signal intensity of background ROI	N/A	‐ Diameter/fluorescence intensity over time ‐ Contraction frequency ‐ Valve position	Diameter/fluorescence intensity at manually selected single cross‐sections	~500 µm visualized at one time	512 × 512 pixels
Liao et al. (2014)^19^	Mouse popliteal lymphatics	Injection of 2% FITC‐dextran into footpad	Custom MATLAB script	Edge‐finding algorithm	N/A	‐ Diameter over time ‐ Contraction frequency ‐ Valve position ‐ Can add GFP‐tagged cell labels	Continuous mean diameter along the length	~250 µm visualized at one time	1344 pixels length
Moriondo et al. (2013)^28^	Rat diaphragmatic lymphatics	Intraperitoneal injection of FITC‐dextran and microspheres	Image J “diameter” plug‐in	Thresholding of a single line of pixels	N/A	‐ Diameter over time	Manually selected single cross‐sections	7.81 × 6.55 mm	Pixel size 8.85 µm
*Transmitted light intravital microscopy*									
Sarimollaoglu et al. (2018)^31^	Rat mesenteric lymphatics	Bright field—no contrast agent	Custom MATLAB script	Automated edge detection at nominated points	Yes	‐ Diameter over time ‐ Flow velocity ‐ Valve position	Diameter at manually selected single cross‐sections	545 × 545 µm	Pixel size 1.06 µm
Kassis et al. (2012)^24^	Rat mesenteric lymphatics	Bright field/ingestion of fluorescent fatty acid analogue BODIPY FL C_16_	Custom MATLAB script	Diameter tracking algorithm after initial manual selection of wall	Yes	‐ Diameter over time ‐ Flow velocity ‐ Valve position ‐ TG conc (by fluorescence intensity)	Diameter at manually selected single cross‐sections	~1.5 mm visualized at one time	640 × 480 pixels
Akl et al. (2011)^23^	Rat mesenteric lymphatics	Phase contrast—no contrast agent required	Custom MATLAB script (Dixon et al. 2007)	Diameter tracking algorithm after initial manual selection of wall	Yes	‐ Diameter over time ‐ Flow velocity ‐ Valve position	Diameter at manually selected single cross‐sections	~307 × 307 µm	512 × 512 pixels
Doppler optical coherence tomography									
Blatter et al. (2018)^26^	Mouse popliteal lymphatic	None required, but enhanced signal with injection of Intralipid into footpad	Custom MATLAB script	Automated using a 2‐dimensional snake algorithm	N/A	‐ Diameter over time ‐ Flow velocity ‐ 4D valve dynamics	Cross‐sectional area at manually selected single points	~2.1 × 1.4 mm	Resolution of 6 μm axial and 11 μm transverse

Note

Selected papers from each of the most common methodologies and software packages are presented.

Abbreviations: FITC, fluorescein isothiocyanate; GFP, green fluorescent protein.

The vast majority of mesenteric lymphatics are embedded in mesenteric fat, and therefore, methods that rely on sections of lymphatics that are relatively fat‐free (i.e., transmitted light microscopy) are unlikely to truly represent mesenteric lymphatics. The relatively short section measured may not be representative of the whole vessel, or of the range of vessels in that section of mesentery. Furthermore, the need for relatively fat‐free lymphatics limits the use of obese animal models and greatly limits the clinical potential, as human mesentery contains relatively more fat and is much thicker (may exceed 0.5–1 cm in thickness), although there is of course a limit to the amount of fat present before adequate visualization of the lymphatic is lost. Stereomicroscopy with a tracer dye is more adapt at dealing with moderate amounts of mesenteric fat than transmitted light intravital microscopy. However, transmitted light without a tracer offers other important advantages (high spatial resolution, ability to measure flow and valve movement, and no contrast agent), suggesting that the two approaches are complementary, and the optimal imaging modality will depend on the application.

The vmeasur software also allows for extensive measurements of the differences in contractility along an extended length of vessel, a feature lacking in previously described tools. We found that the contraction amplitude along the length of a single lymphatic vessel was highly variable, which has also been shown in diaphragmatic lymphatics.^28^ This variability did not seem to represent differences in contractility between the central section of a lymphangion and the valve area because it was on a larger scale (up to 1 cm in Video S1) than the typical length of a mesenteric lymphangion (less than ten times the diameter,^32^ that is, 0.8–2 mm for the vessel in Figure 2). Seemingly simultaneous contractions across valve regions have also been observed elsewhere,^27^ further suggesting that the spatial heterogeneity demonstrated is not based on the distribution of lymphangions. We also found that adjacent lymphatic vessels can be similarly variable in their contractile function (data not shown). This highlights the need to capture contractile parameters over an extended section of a lymphatic vascular tree.

The vmeasur software measures the spatial variation in contractile function by splitting the ROIs into equal‐length sections. This allows for quantitative, simultaneous comparisons between different sections of the same vessel, multiple vessels from across the mesentery or multiple subjects. The strength of this standardized length approach is highlighted by considering one long vessel that has a large contraction over a small section and a second vessel that undergoes a small contraction over a longer length of vessel. If the parameters of the whole vessel were averaged, these two scenarios would have similar contraction parameters, despite these representing different physiological contractions with different effects on flow. Although looking at each individual row of pixels would be ideal, these data quickly become excessively noisy due to limitations in image acquisition. By selecting a mid‐range length of physiological relevance, data can be generated that represents the variation along the length without excessive random noise. Hence, vmeasur may allow for novel insights not possible with existing software.^19^

Using this approach, a large amount of data can be efficiently generated from a 30 s video of a single lymphatic vessel. If this process is repeated for longer timeframes and more sites within the mesentery, the amount of data generated becomes substantial. Despite this scale, vmeasur can handle these datasets by combining image manipulation, video processing, and parallel processing packages into a single workflow. This allows almost the entire rodent mesentery to be assessed within manageable timeframes using standard computing resources. Video stabilization and/or image registration to remove any movement of the lymphatic vessel due to breathing and nearby arterial pulsations, which is required in most other software platforms,^24^ is not required by vmeasur due to the threshold‐based segmentation method and wider field of view. This efficient quantification further simplifies and accelerates data acquisition.

The wide field of view combined with the rapid surgical technique and image processing allows one to sample a large number of mesenteric lymphatics, offering a more accurate representation of overall mesenteric lymphatic contractile function. This contrasts with transmitted light intravital microscopy, which, in relation to the length of vessel measured, is time‐consuming to set up. Because lymphatic contractility is so variable (either due to intrinsic lymphatic factors,^33^ variability in pressure or flow,^4^ or the local tissue microenvironment^34^), a large sample size is necessary to obtain a true representation of lymphatic function in any one animal. This is vital when comparing lymphatic contractility between different groups or when testing pharmacological agents that are administered systemically. In diseased animals whose condition may degrade rapidly, it is important to obtain as much information as quickly as possible. Furthermore, the rapid setup and existing ubiquity of stereomicroscopes lends itself to clinical utility, where intra‐operative assessment of mesenteric lymphatic contractility is likely to be time‐sensitive.

Vmeasur includes highly automated tracking and contraction detection processes to allow for rapid analysis while minimizing user bias. To ensure that this automated analysis is accurate, the edges of the vessel and graphical representations of the contraction event are displayed for real‐time manual validation. While the automatic threshold is generally correct, there is the option for the user to intervene whether there are any issues. Most thresholding failures occur when the ROI is incorrectly drawn so that it encompasses blood vessels or areas of excessive light reflection. In these situations, it is often better to revise or exclude the ROI rather than manually adjust the threshold as the operator may inadvertently introduce bias. Thresholding may also fail in diseased animals that show some extravasation of blue dye due to increased lymphatic vessel permeability. Here, the user can manually increase the threshold until it detects the true vessel edge. Generally, it was found that automated contraction event detection reliably detects true lymphatic contractions. This automation greatly speeds up the analysis of large datasets, when compared to methods which require a user‐defined threshold (e.g., Liao et al.^19^). Other existing software does not include automatic detection of end‐systolic and EDD, and subsequent automatic calculation of contraction frequency, and instead requires the user to manually calculate these parameters.

### 4.1 Applicability

This wide‐ranging applicability of our software package and image acquisition technique is important, as mesenteric lymphatic contractile dysfunction is becoming increasingly recognized in many disease states. Dysfunctional contractility is known to contribute to bowel wall inflammation^14^ and decreased contractility has been demonstrated in animal models of peritonitis^35^ and ileitis.^36^ Mesenteric lymphatic contractile dysfunction has also been detected in systemic disorders, such as obesity.^37^ This technique will facilitate investigation and research of mesenteric lymphatic contractility in different animal models, including what happens in the context of acute and critical illness. While we did not have the opportunity to analyze a video recording of a human mesenteric lymphatic, we were able to show the potential clinical utility of this method on an intra‐operative human image (Figure 4). The rapid and straight‐forward nature of this technique, which is not reliant on fluorescence, transmitted light, or areas of mesentery devoid of fat, lends itself to clinical applicability, which has significant implications for further research on *in vivo* human mesenteric lymphatic contractility.

### 4.2 Limitations

A key limitation of this software package is the need to use contrast‐enhanced images captured after injection of dye into the bowel wall. Although the volume needed is small, there is an inevitable minor amount of tissue damage. Injecting a volume of fluid also affects the interstitial pressure driving lymph formation. An increase in intraluminal lymphatic pressure is known to increase contraction frequency and amplitude over a certain range and an increase in lymph flow is known to decrease contraction frequency.^4^ This problem can be controlled for by injecting the same (or similar) volume into the different groups that are being compared, although this will still not guarantee consistent lymphatic pressures and flow.^18^ Because this technique is designed for comparing lymphatic contractile function across healthy and disease groups, any small inconsistencies in filling pressure should be overcome with sufficiently large sample sizes. This method lends itself to larger sample sizes, as it allows for more efficient collection of data from multiple sites compared to conventional intravital microscopy. The type of dye should also be considered and kept constant across groups. Zawieja et al.^18^ presented evidence that Evan's blue dye completely stopped spontaneous contractions in *ex vivo* peripheral mouse lymphatics when injected distally prior to removal. We did not find this effect *in vivo* and have noticed normal spontaneous contractions in the rat mesentery when using Evan's blue, but care should be given when comparing to other studies that use a different dye. Furthermore, the reduced resolution compared to transmitted light microscopy does not allow for direct measurements of lymph flow or valve position. The latter has implications for the calculated fractional pump flow, which will be inaccurate if any valvular incompetence is present. The lower resolution is also evident in Figure 2D where vmeasur determined that the diameter contracted down to zero in places, despite this being unlikely in reality. However, again this is less important when comparing between groups when the same analytical method is used. Lastly, although we used a body temperature‐controlled heating pad to maintain body temperature at 37 °C and perfused the mesentery with warmed normal saline during imaging, which has also been done in other studies,^26^, ^27^, ^38^ using a temperature‐controlled perfusion bath would be ideal. However, as transmitted light is not required and hence opaque items can be placed directly under the sample, introducing a perfusion bath or other further equipment would not affect the performance of the software.

## 5 CONCLUSION

As mesenteric lymphatic contractility is becoming increasingly important in a range of diseases, there is the need for laboratories to have access to a reliable, rapid, and inexpensive technique to assess contractile function. The imaging approach and subsequent analysis technique presented here will prove useful in many contexts and have distinct advantages over current imaging approaches and software packages. These include the ability to measure lymphatic contractile parameters over relatively long lengths of lymphatic vessels that are embedded in mesenteric fat and simultaneously compare the contractile parameters along the length of a vessel and between adjacent vessels. This is important because of the wide variability in contraction parameters between different vessels and different sections of the same vessel, which has been demonstrated in this study. Furthermore, because lymphatic contractility can be determined in vessels embedded in fat, this method has potential for clinical use. The limitations of this method, including the use of a contrast agent, make it complementary to other well‐established techniques such as transmitted light intravital microscopy.

## 6 PERSPECTIVE

Mesenteric lymphatic contractile dysfunction is becoming increasingly recognized as having a role in a variety of conditions, but software for objective and clinically translatable measurement of this lymphatic function under low magnification has been lacking. This paper describes a freely available R package (“vmeasur”) that was shown to measure mesenteric lymphatic contractions over an extended field of view in a rat. The software also has potential for clinical use.

## CONFLICT OF INTEREST

NT is an inventor of a lymph‐directing glyceride prodrug technology, which enhances delivery of drugs to intestinal lymph. This technology has been patented and licensed via a commercial agreement with PureTech Health, Boston. PureTech Health have subsequently entered into a collaboration agreement with Boehringer Ingelheim to explore the technology in immune modulation. NT receives payments and royalties from PureTech Health as part of the agreement.

## Supporting information

Video S1

Video S2

Fig S1‐S2
