# Supplementary material for: Vmeasur: A software package for experimental and clinical measurement of mesenteric lymphatic contractile function over an extended vessel length
Source: Microcirculation. 2022 Feb 10;29(6-7):e12748. doi: 10.1111/micc.12748 (PMC9787391; doi:10.1111/micc.12748)
Supplement: Supplementary file 3 — Fig S1‐S2 [file MICC-29-e12748-s001.docx]

**SUPPLEMENTARY MATERIAL**


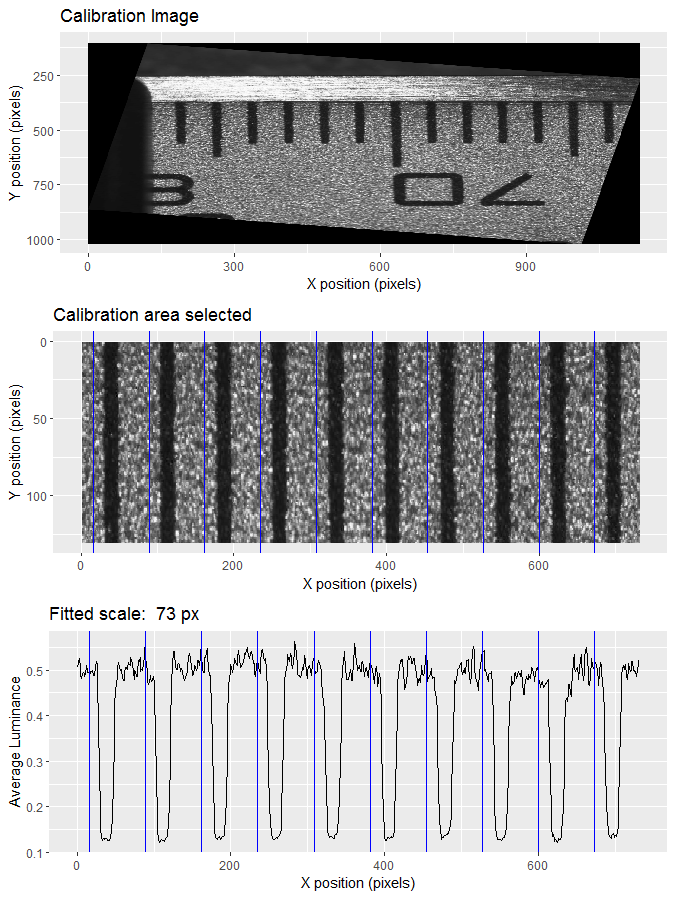


Figure S1: **Procedure to calibrate the pixel size of a dissecting microscope.** As the focal length of dissecting microscopes is fixed at a particular magnification, the level of zoom is also constant. However, this zoom cannot be directly converted into a number of pixels per millimeter as this value will be affected by the exact configuration of the microscope and camera. Therefore, vmeasur includes a tool to calibrate the size of each pixel in a microscope image. Firstly, an image of a ruler graded in mm should be acquired using the camera and microscope that will be used for further experiments. By calling the calibrate_pixel_size function in vmeasur, the user is presented with a dialog box where they can select this image from their computer. The user is then prompted to draw a line along the length of the scale. This line is used to rotate the image so that the scale is perfectly horizontal. A second prompt allows the user to select a section of the rotated image that contains only the gradations, whilst excluding any numbering, annotation or other features of the ruler used. vmeasur will then average the pixel intensity for each column of pixels. This results in a series of numbers that represent the luminance of the scale across the width of the image. By running autocorrelation analysis using the acf function, the cyclic nature of the luminance intensities, and hence the distance between gradations, can be determined. Offsets where the calculated autocorrelation crosses the x axis are determined to be single gradations. To allow for manual review, these values are plotted as vertical lines on top of the luminance values previously determined and superimposed on the original image. The mean distance between each of the gradations is then calculated, and taken to be the number of pixels/mm. This value can then be used to convert pixel measurements to absolute distances in mm. The microscope we used had 73 pixels/mm.


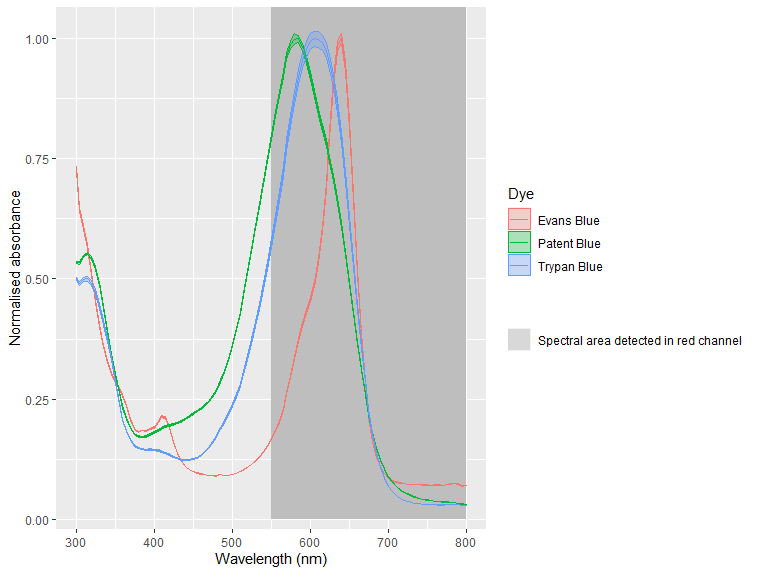


Figure S2: **A variety of blue dyes are detectable using the red channel in CCD cameras**. The spectra of solutions of 1% w/v Evans Blue, 2.5% w/v Patent Blue VF and 0.4% w/v Trypan Blue were measured. The wavelengths detectable with a standard CCD camera were then overlaid. The graph shows the absorbance mean normalized to the highest absorbance ± SEM of three readings.
